# Zur Zukunft der EU-Finanzen nach Corona

**DOI:** 10.1007/s10273-020-2799-8

**Published:** 2020-12-26

**Authors:** Michael Broer, Klaus-Dirk Henke, Horst Zimmermann

**Affiliations:** 1 Fakultät Wirtschaft - Campus Wolfsburg, Ostfalia Hochschule für angewandte Wissenschaften, Siegfried-Ehlers-Str. 1, 38440 Wolfsburg, Deutschland; 2 Institut für VWL und Wirtschaftsrecht, Fak. VII, TU Berlin, Fraunhoferstraße 33-36, 10587 Berlin, Deutschland; 3 Koenigsberger Str. 17, 35043 Marburg, Deutschland

**Keywords:** F3, F15, F55, H77, H81, H87, N44

## Abstract

Im Zuge der Corona-Krise erfuhr die Finanzierungsgrundlage der EU größere Änderungen, die in der Frage resultieren, welche Form die zukünftige Finanzverfassung der EU annehmen wird. Der zusätzliche Wiederaufbaupakt mit einem Volumen von drei Vierteln des neuen mehrjährigen Finanzrahmens wird durch die erstmalige Verschuldung der EU selbst finanziert. Reicht dieses beispiellose fiskalische Volumen, um zukünftig im Wettbewerb mit China bestehen zu können? Oder braucht die EU eine Neuausrichtung: zum einen in Bezug auf Umfang und Differenzierung ihrer verschiedenen Aufgaben und zum anderen bezüglich der gleichen Rechte ihrer 27 Mitgliedstaaten? Die Umbrüche infolge der Corona-Krise sollten für eine größere Reform der EU-Finanzen genutzt werden.

## Abstract (en)

The impact of COVID-19 led to changes in EU financing that pose questions about the shape of the future EU “fiscal constitution”. The additional Recovery Fund, with the volume of three-fourths of the new seven year budget, will be financed by the first EU borrowing ever. Is this unprecedented fiscal magnitude necessary to compete with China? Or does the EU need to downsize in two directions: (1) in the volume and differentiation of its various tasks and (2) in the equal rights among its 27 member states (01.01.2021). In any case, the COVID-19 shakeup should be incentive for a major reform of the EU finances.

Im Vergleich internationaler Organisationen ist die Integration souveräner Staaten in der EU am weitesten fortgeschritten, wobei der Umfang und die Struktur des EU-Budgets meist im Vordergrund stehen. Diese Situation hat sich angesichts der Corona-Krise erheblich verändert. Die Kommission hat „auf Ersuchen der Staats- und Regierungschefs ein sehr weitreichendes Paket vorgelegt, in dem der künftige mehrjährige Finanzrahmen (MFR) und spezifische Aufbaumaßnahmen im Rahmen von ‚Next Generation EU' (NGEU) miteinander verknüpft werden" (Europäischer Rat, 2020). Dieses Bild wird kontrovers diskutiert, da unklar ist, welches Stadium die EU erreicht hat und erreichen soll. Befindet sich die EU auf dem Wege in eine Fiskalunion, oder ist mit diesem Begriff schon ihr Zustand beschrieben? Mit Blick auf diese Ausgangslage können zwei Auffassungen über die wünschenswerte Zukunft der EU unterschieden werden:

- Ein Staatenbund als ein Zusammenschluss souveräner Staaten mit eigener Organisation auf der Zentralebene: Völkerrechtlich handelt es sich dabei um eine Staatenverbindung. Sie verfügt weder über ein eigenes Gebiet noch über eigene Staatsangehörige. Ein Staatenbund selbst ist kein Staat.

- Ein Bundesstaat als ein Staat, der sich aus mehreren Teil- oder Gliedstaaten zusammensetzt: Rechtlich besteht ein Bundesstaat aus mehreren politischen Systemen mit Staatsqualität. Er vereint eine Bundesebene und mindestens eine Ebene der Gliedstaaten. Damit unterscheidet sich dieser föderal organisierte Staat sowohl von einem locker gefügten Staatenbund als auch von einem zentralistischen Einheitsstaat.

Dementsprechend sehen manche Mitgliedstaaten die EU als eine lockere Gruppe von Staaten mit einigen gemeinsamen Institutionen, andere gleichsam als eine unvollständige Föderation. Die Realität und die deutsche Position passen bislang mehr zu der ersten Gruppe, während die zweite Gruppe vielleicht hinter manchen aktuellen Vorschlägen zur Reform der EU steht.

Allerdings haben die Gliedstaaten auf vielen Gebieten der EU das Recht übertragen, für alle Staaten gemein-

**Prof. Dr. Michael Broer** ist Professor für Volkswirtschaftslehre an der Ostfalia Hochschule für angewandte Wissenschaften in Wolfsburg.

**Prof. em. Dr. Klaus-Dirk Henke** ist Professor für Finanzwissenschaft und Gesundheitsökonomie am Institut für Volkswirtschaftslehre und Wirtschaftsrecht an der TU Berlin und Mitglied des Wissenschaftlichen Beirats beim Bundesministerium der Finanzen.

**Prof. em. Dr. Horst Zimmermann** war Professor für Finanzwissenschaft an der Philipps-Universität Marburg und Mitglied des Wissenschaftlichen Beirats beim Bundesministerium der Finanzen.

sam verbindliche Regeln zu schaffen, die den Regelungen der Einzelstaaten vorgehen. Genannt seien beispielhaft verbindliche Regelungen für den Wettbewerb, die Umsatzsteuer, Niederlassungsfreiheit, Reisefreiheit oder die Gewerbefreiheit. Diese für einen gemeinsamen Binnenmarkt und das tägliche Leben aller Bürger*innen wichtigen Bestimmungen führen noch nicht zu einem gemeinsamen Bundesstaat. Die EU-Länder sind aber sicher schon mehr als ein Staatenbund.

### Die Zukunft der Finanzverfassung der EU

Angesichts der mit der Corona-Krise einhergehenden neuen fiskalischen Gegebenheiten, wie den anstehenden Entscheidungen über das EU-Budget 2021-2027 und dem Wiederaufbaupakt (Recovery Fund), nimmt die Komplexität der Ausgangslage deutlich zu. Die Lage war bisher durch einen EU-Haushalt in einem mehrjährigen Finanzrahmen gekennzeichnet, der – abgesehen von den Zolleinnahmen – überwiegend über die Beiträge der Mitgliedstaaten finanziert wurde. Jetzt haben die Mitgliedstaaten mit der Entscheidung des Gipfels Ende Juli 2020 in gewissem Sinne eine neue Finanzverfassung geschaffen. Sie rücken die EU-Finanzen in dem genannten Spektrum zwischen Staatenbund und Bundesstaat ein Stück weiter weg vom Staatenbund. Jedenfalls lässt sich zwischen dem Status quo ante, also vor den Beschlüssen der Sondertagung des Europäischen Rates (17. bis 21. Juli 2020), und der danach beginnenden Zeitachse bis zum Jahr 2058 unterscheiden, wenn die von der EU für gemeinsame Rechnung aller Mitgliedstaaten aufgenommenen Schulden endgültig getilgt sein sollen.

Bundesfinanzminister Scholz geht davon aus, dass es sich bei der Schuldenaufnahme nicht um einen einmaligen Schritt, sondern um eine strukturelle Veränderung der EU-Finanzierung handelt (Scholz, 2020). Anders hingegen die Sicht der Bundeskanzlerin, verdeutlicht durch ihren Sprecher (o. V. 2020), der die Schuldenaufnahme mit der außergewöhnlichen Situation begründete und darauf hinwies, dass die Verschuldung zeitlich und auch vom Umfang her begrenzt ist.

In dieser Diskussion über die Finanzverfassung der Europäischen Union spielen viele Aspekte eine Rolle, die unterschiedlich eingeschätzt werden (Broer und Henke, 2020) und auf der Grundlage der Beschlüsse des Europäischen Rates im europäischen Parlament auch noch revidiert werden können.

- Es beginnt mit dem Gewicht, das die EU in einer unsicheren Zukunft zwischen den Blöcken USA und China einnehmen soll, und vor dem Hintergrund, dass sie zu selten mit einer Stimme spricht. Ist eine EU mit einem Wiederaufbaupakt in Höhe von 750 Mrd. Euro (neben ihrem Sieben-Jahres-Budget) gut genug gewappnet, um die großen Aufgaben wie die Corona-Krise, den Klimawandel, die Digitalisierung und das Flüchtlingsproblem in Zukunft effektiv und effizient zu lösen? Wer den europäischen Bundesstaat im Auge hat, wird das verneinen und mehr verlangen. Wer die EU als Staatenbund sieht, wird auf die fehlende Durchsetzungsstärke bei 27 Mitgliedstaaten verweisen und sich vielleicht auf Staaten wie Frankreich und Deutschland verlassen. Mit dem Brexit hat mit Großbritannien das neben Frankreich einzige wirklich wehrfähige und wehrwillige Mitglied die EU verlassen. Wie tritt Europa in diesem Kontext weltweit am besten gegenüber anderen Weltmächten auf?

- Bei der Art der von der EU zu erfüllenden Aufgaben sollten daher diejenigen mit europaweitem Nutzerkreis im Vordergrund stehen (Henke, 2020). Dementsprechend müssten die Agrarausgaben, die einen Großteil des Budgets verschlingen aber nur einigen wenigen Mitgliedern zugutekommen, zurückgeführt werden. In einem dezentralen Staatenbund werden dessen Aufgaben nicht nur häufiger hinterfragt, sondern auch in ihrem Grad an Detailvorschriften eingeschränkt. Bemängelt wird daher nicht nur die zunehmende Kompetenzaneignung der Europäischen Kommission, also der Exekutive, sondern auch die damit verbundene mangelhafte Wahrung des Subsidiaritätsprinzips (Henke, 2006).

- Auf der Einnahmenseite hat sich durch die Beschlüsse zur Finanzierung des Wiederaufbaupakts die Situation stark verändert. Die Schuldenaufnahme durch die EU-Kommission deutet insoweit auf einen Schritt in Richtung einer Fiskalunion hin, da bei den aufgenommenen Schulden nicht der Einzelstaat, sondern die EU insgesamt haftet. Hinter der EU stehen die 27 Einzelstaaten und diese haften entsprechend ihrer Beiträge, die an der wirtschaftlichen Stärke orientiert werden. Der Umgang mit der hohen Staatsverschuldung ist ein besonderer Streitpunkt (Paqué, 2020). Wird eine Bundesstaatslösung angestrebt, so ist die Ausgabe von Eurobonds eine geeignete Maßnahme. Denn hier profitieren die hoch verschuldeten Staaten, weil sie für sich allein nur zu ungünstigen Konditionen Anleihen aufnehmen können. Nicht nur Deutschland verweist aber auf die langfristigen (Fehl-)Anreize einer solchen Maßnahme. In einer Schuldenunion wäre das Haftungsrisiko der bereits stark verschuldeten Staaten verringert, und damit verbunden würde sich der Druck auf die Schuldnerländer mindern, ihre Volkswirtschaft durch Konsolidierungsmaßnahmen zu stabilisieren und damit ihre Schuldenstandsquote zu verringern. Diese Argumentation zielt eher in Richtung Staatenbund.

- In der Corona-Krise mit ihren ungewöhnlich hohen finanziellen Belastungen tritt dieser Streitpunkt kurzfristig zurück, da die erforderliche Hilfe für die besonders notleidenden südlichen EU-Staaten statt der gelegentlich geforderten Corona-Bonds[1] zu einem Teil die Form von direkten Transfers (verlorene Zuschüsse) annimmt.[2] Insbesondere die Niederlande und Österreich haben sich besonders vehement gegen diese finanziellen Hilfen ausgesprochen. Da die Rückzahlung der Schulden bis zum Jahre 2058 vorgesehen ist, lässt sich nicht ausschließen, dass in dieser langen Periode die oft als Schuldenunion bezeichnete EU allmählich ergänzt wird durch eine „Solidarunion", die auch, wie zumindest geplant, über eigene Steuern verfügen wird.

## Die Corona-Krise als Zäsur

Sieht man den europäischen Einigungsprozess nicht nur als Verlängerung seiner vielleicht in verschiedenen Zusammenhängen zu beklagenden Krisengeschichte an, dann könnte „an die Stelle eines unspezifischen, unumkehrbaren Integrationsprozesses ein projektgebundenes föderatives Handeln treten. Die Corona-Pandemie wäre dafür ein guter Anlass" (Kielmansegg, 2020; Enderlein, 2020). Auch Wolfgang Streeck (2020) sucht nach neuen Wegen für die EU. Mit seinem „Vorschlag einer begrenzten, nach gemeinsam ausgewählten Aufgabenfeldern gestalteten Europäischen Union" zeigt er in die gleiche Richtung: Die treibenden Kräfte, welche die Corona-Pandemie ausgelöst hat, wären insoweit zu nutzen, um nicht nur die Gefahren der Krankheit und eines neuen Lockdowns zu bewältigen. Vielmehr könnte die Corona-Pandemie zugleich zur Zäsur für einen Neuanfang der EU werden. „Dort wo die EU handlungsfähiger werden will, muss sie sich konzentrieren auf diejenigen Bereiche, in denen die Union einen echten Mehrwert für die Mitgliedstaaten erbringt: Die Bereiche hierfür sind unter anderem Digitalisierung, Netzinfrastruktur und Verkehrswege, Asyl- und Migrationspolitik, Sicherheits- und Verteidigungspolitik und ein Neustart der Währungsunion" (Rödder, 2020).

Wenn es um die Bereitschaft der Mitgliedstaaten geht, in der EU stärker zusammenzuarbeiten, können auch die schon früher vorgelegten Vorschläge zu einem „Europa der zwei Geschwindigkeiten" erneut aufgegriffen werden. In den bisher erörterten Vorschlägen bleiben Zahl und Gewicht der Mitgliedstaaten in der Regel unverändert. Wem die Vorschläge zur Stärkung der EU zu weit gehen, wird aber auf die Schwierigkeit hinweisen, mit 27 Mitgliedstaaten von Frankreich und Deutschland einerseits bis Malta und Zypern andererseits schnell erforderliche und wichtige Beschlüsse zu fassen.[3] Er wird möglicherweise darauf verweisen, dass die ersten Anleihen zur Bekämpfung der Corona-Krise erst 2021 fließen, und wird den Vergleich mit den oben beschriebenen schnellen deutschen Maßnahmen ziehen. Um das auch auf EU-Ebene zu ermöglichen, ist aus dieser Sicht vielleicht ein „Europa der zwei Geschwindigkeiten" anzustreben. Innerhalb der Eurozone sind bereits vor der Corona-Krise Überlegungen zu einem Nord-Euro entwickelt worden (Zimmermann, 2015). Auch für die EU als Ganzes sei auf die im Frühjahr 2020 angestrebte „Koalition der Willigen" für Flüchtlingskinder verwiesen, innerhalb derer eine Abstimmung leichter ist als zwischen allen Mitgliedstaaten.[4] Auch könnte für die EU als Ganzes ein engeres deutsch-französisches Zusammengehen ein guter Weg sein, wie ihn Cohn-Bendit und Leggewie (2020) skizziert haben. Eine weitere Reformmöglichkeit bestünde darin, die Abstimmungsregelungen der EU im Ministerrat weiter zu verändern und auf mehr Entscheidungen mit qualifizierter Mehrheit zu setzen.

Vor diesem Hintergrund könnte die Corona-Krise tatsächlich einen Schub für ein stärkeres Europa auslösen, in dessen Mittelpunkt das EU-Budget steht. Allerdings ist eine Stärkung des europäischen Parlaments dafür ebenso unabdingbar wie eine klare europäische Rechtsstaatlichkeit. Grundsätzlicher sind allerdings die Vorschläge zu einer stärkeren Legitimierung der EU-Kommission und damit der Exekutive durch das europäische Parlament, das mit Budgetrechten ausgestattet werden müsste (Müller und Richter, 2020). „No Europe without the rule of law" (James, 2020).

Wenn man die getroffenen Entscheidungen zur zukünftigen Finanzierung akzeptiert, geht es nunmehr um die Umsetzung der gut 60-seitigen Schlussfolgerungen der außerordentlichen Tagung des Europäischen Rates vom Juli 2020. Zur Vergabe der Mittel durch die EU-Kommission lassen sich Kriterien (Europäischer Rat, 2020) und Fragen (Kafsack und Mussler, 2020; Mussler, 2020) heranziehen, die bei der Verwendung der Mittel aus dem „Recovery Fund" dienen können. Hierzu bedarf es allerdings

1 Corona-Bonds sind Wertpapiere mit einem festen Zinssatz. Die europäischen Staaten könnten zusammen solche Anleihen an den Markt bringen. Die Regierungen würden auf diesem Weg gemeinsam Geld an Finanzmärkten aufnehmen, sich also verschulden – und dann gemeinschaftlich für Zinsen und Rückzahlung haften.

2 So Thum und Weichenrieder (2020) schon vor der EU-Beschlussfassung.

3 Zu den Schwierigkeiten bei der Abstimmung schon zwischen drei Entscheidungsträgern siehe Zimmermann et al. (2017).

4 Zum Vergleich verschiedener Vorgehensweisen siehe Dilger (2018).

neuer Formen und organisatorischer Formate, welche die Effektivität und Effizienz der verwendeten Mittel in innovativer Form sicherstellen.

Nicht zuletzt machen die Infektionszahlen deutlich, dass neben den wirtschaftlichen Rahmenbedingungen vor allem die gesundheitlichen und sozialen Folgen der Corona-Pandemie hoch auf der politischen Agenda stehen müssen. Europaweit ist hier mehr Solidarität unter den Mitgliedsländern erforderlich. Mehr Solidarität heißt aber nicht zwingend mehr und größere Geldtransfers, sondern sollte auch in von den Bürger*innen wahrnehmbarer sonstiger Hilfe bestehen wie z. B. Katastrophenschutz, Know-how-Transfers bei organisatorischen Fragen, bessere Zusammenarbeit von Polizei, Zoll, Steuerbehörden, Universitäten etc.
